# Virtual reality and its use in post-operative pain following laparoscopy: a feasibility study

**DOI:** 10.1038/s41598-022-17183-2

**Published:** 2022-07-30

**Authors:** Olivia Payne, Vinayak Smith, Daniel L. Rolnik, Miranda Davies-Tuck, Ritesh Warty, Densearn Seo, Lima Wetherell, Hamsaveni Kalina Murday, Amrish Nair, Rashvinder Kaur, Beverley Vollenhoven

**Affiliations:** 1 Department of Obstetrics and Gynaecology, Monash University, 246 Clayton Road, Clayton, VIC 3168 Australia; 2 Monash Women’s and Newborn Program, Monash Health, 246 Clayton Road, Clayton, VIC 3168 Australia; 3 Biorithm Pte Ltd, 81 Ayer Rajah Crescent 03-53, Singapore, 139967 Singapore; 4 Hudson Institute of Medical Research, 27-31 Wright Street, Clayton, VIC 3168 Australia; 5 Central Clinical School, Faculty of Medicine, Nursing, and Health Science, Monash University, 99 Commercial Rd, Melbourne, VIC 3004 Australia

**Keywords:** Pain management, Surgery, Biomedical engineering

## Abstract

Pain following laparoscopic surgery remains a neglected healthcare issue. Virtual reality-mediated therapy’s (VRT) analgesic potential could address this. However, its effect in this setting remains unexplored. We aimed to establish the feasibility and safety of VRT as an adjunct analgesic following gynaecological laparoscopy and explore differences between active distraction and passive meditation content. 35 women were enrolled into an open crossover pilot and randomised to either intervention group 1 (active then passive content) or intervention group 2 (passive then active content) following surgery. VRT was administered in two 10-min segments with a 10-min washout period in between. Pain scores, opioid requirements and side effects were recorded before and after each segment whilst questionnaires evaluated acceptability. We observed a significant reduction in pain over time for the entire study population (F = 8.63, p < 0.0005) but no differences between intervention groups, in contrast to many studies demonstrating an increase in pain during this time. During segment one, intervention group 1 (n = 18) were administered significantly less opioid than intervention group 2 (n = 17) [0.0 (0.0–7.5) vs. 3.0(0.0–10.0), p = 0.04]. Intervention group 1 rated the VRT experience significantly higher than intervention group 2 (7.97 vs. 6.62. p = 0.017). 97.1% (n = 34) would recommend VRT to a friend and use it as the standard-of-care in future procedures. These results demonstrate that post-operative VRT is feasible and safe. However, adequately powered studies are needed to appropriately determine its efficacy.

## Introduction

Post-operative pain (POP) is a clinical phenomenon that can affect between 20 and 40% of patients^1,2^. Even minimally invasive (MI) procedures such as laparoscopy are afflicted by POP, with 35–65% of patients undergoing such operative procedures reporting clinically significant pain levels, with some suggestion that this may even supersede the pain experienced during laparotomy^3,4^.

At present, opioids remain the mainstay of treatment for POP. However, opioids have several widely acknowledged shortcomings such as adverse side effects, delayed ambulation and the potential for dependence^5,6^. As such, there remains an urgent need to characterise adjunct analgesics to improve the clinical management of POP, reduce the reliance on opioids and importantly, improve patient outcomes^7,8^.

Virtual reality (VR) is a burgeoning technology that offers the potential to bridge this gap in analgesia by immersing users in a virtual environment (VE)^9–12^. To do this, either active or passive content types can be utilised. The distinction between the two lies in the level of interaction as passive content merely involves observing the VE whilst active content requires participation.

Recently, a meta-analysis demonstrated the ability of VR-mediated therapy (VRT) as an analgesic in the setting of POP following both minor and major surgery (haemorrhoidectomy, dental surgery, episiotomy repair, craniotomy/spine surgery and knee surgery), reporting a significant decrease in pain scores in this setting^13^. These findings were echoed by a recent systematic review demonstrating the efficacy of VR in reducing pain within inpatient populations for various pathologies, including: hydrotherapy for burns/wounds; venepuncture; and injections^14^. Tashjian et al. further demonstrated this within an inpatient setting, reporting that VRT, in comparison to control (2D video of nature scenes), led to a significant reduction in acute pain (24% vs 13.2%, p = 0.008) in hospitalised patients (12% of patients had POP). Moreover, VRT appeared effective at encouraging patients to respond to treatment as well (number needed to treat = 4)^15^. Importantly, these findings appear to be associated with low to negligible side effects and an improvement in patient experience^13,14,16,17^.

Although promising, a number of questions regarding the utility of VRT in the immediate post-operative (PO) period following laparoscopy remain unanswered. Primarily, there is no information regarding the feasibility, acceptability and safety of VRT for this indication, as studies to date have neither been conducted post-abdominal-surgery nor assessed patients within the immediate peri-operative period (within 2 h of surgery)^13^. Additionally, despite experimental evidence suggesting that active VR has a greater analgesic effect than passive VR, this is yet to be clinically validated/investigated in the context of POP^9,18,19^.

### Aims

To address these gaps in knowledge, we carried out a proof of concept pilot study in patients with POP following MI gynaecological laparoscopy. The aims of this study were to compare the effect of active distraction and passive meditation VRT on post-operative pain, opioid requirements and adverse outcomes in women undergoing laparoscopy. Additionally, the study aimed to determine the feasibility of VRT following laparoscopic surgery.

## Methods

### Study design

The study was an open-label single-centre randomised crossover pilot trial in 35 women in a tertiary university teaching hospital, between April and August 2019. This trial had approval from the Monash Health Human Research and Ethics Committee (HREC/45131/MonJ-2018-150802) and was performed in accordance with the Good Clinical Practice principles. This trial was also registered on the Australia-New Zealand Clinical Trials Registry (ACTRN12618001398291p; registration date: 20/08/2018).

### Participants

Women were eligible for inclusion in this study if they were above 18 years of age and undergoing a laparoscopic procedure for a gynaecological condition.

Clinical exclusion criteria were: conversion to laparotomy; chronic narcotic use or narcotic dependence; pregnancy; patients with an intellectual impairment; patients with co-morbidities such as pre-existing heart disease; and people in existing dependent or unequal relationships with researchers.

Technology-related exclusion criteria were: prior sensitivity to VR technology; motion sickness; vertigo; seizures; epilepsy; and active nausea and/or vomiting. Women were recruited at the time of their attendance at the day surgery centre via convenience sampling.

### Randomisation and blinding

Randomisation for the sequence of active and passive content was carried out via randomised permutated blocks using Microsoft Excel 2016, with block sizes of 10 and a 1:1 ratio at the time of recruitment. Due to the nature of VRT and the fact that each type of content required explanation before administration, the allocation was not concealed from the researcher nor participant. No blinding was implemented in the analysis nor the interpretation of the results considering this was a proof of concept study.

### Outcomes

The main primary outcome of this study was patient-reported pain scores. POP was evaluated using an 11-point numerical rating scale (NRS), which was verbally delivered to participants. This scale was chosen as it demonstrates validity and reliability in a clinical setting^20,21^. The NRS, a 10 cm horizontal line, was defined by the end points: 0 = “no pain at all”; and, 10 = “worst pain I can imagine”. Women were asked to rate their pain by assigning it a number between 0 and 10, based on the above definitions. Patients were evaluated for the pain felt at the specific time points of 0, 10, 20 and 30 min.

As an additional primary outcome, we assessed the feasibility and acceptability of VRT in the immediate post-laparoscopy setting. Feasibility was assessed through recruitment and dropout rates, where it was determined a priori that a recruitment rate ≥ 50% and a dropout rate of ≤ 20% following randomisation would be necessary for the trial to be deemed a success. The outcome of acceptability was assessed through a post-intervention questionnaire aimed at determining patients’ opinions of the device and VRT as an analgesic. Women were asked to rate the experience of using the device, whether they would use it again, recommend it to others as well as other questions (Supplementary Materials).

Secondary outcomes included:

1. Opioid use, defined as the amount of PO opioids administered to patients were recorded at the time of administration by the trial investigator. Later, for analysis, they were converted to morphine sulphate equivalents via the Australian and New Zealand College of Anaesthetists (ANZCA) validated dose equivalence analgesic table^22^. PO opioids were analysed based on the time period in which they were administered (time periods: prior to protocol; between 0–10; 10–20; and, 20–30 min).
2. Adverse outcomes, defined as nausea, active vomiting, dizziness, vertigo, seizures and were evaluated through a questionnaire administered to women at 0, 10, 20 and 30 min. For the trial to be deemed safe, it was determined a priori that the number of participants experiencing side effects must be ≤ 30% under a per protocol analysis. This threshold was determined with consideration to the level of post-operative side effects that may be anticipated within this population which can be a confounding factor due to the similar symptoms with cybersickness. Participants who did not complete the protocol are reported separately.

In addition, a pre-operative questionnaire was administered to patients to gather their demographic data as well as their perception towards pain and anxiety associated with the procedure (Supplementary Materials).

### Study procedures

Eligible patients were approached prior to their surgery by the trial investigator (unrelated to their care), when written informed consent was obtained, and the trial procedure explained.

Participants then underwent their laparoscopic procedure, which was carried out by an experienced surgeon (> 100 procedures per surgeon). The surgical technique was determined at the discretion of the operating surgeon and as per clinical guidelines. Similarly, the anaesthetic care was provided by the anaesthetic team at their discretion. This was chosen to allow the findings to be generalisable to any PO patient, in line with real world evidence.

Following surgery, patients were immediately taken to the post-acute care unit (PACU). The PACU clinician caring for the patient as well as the patient used an 11-point visual analogue scale (VAS) to define the patient’s cognitive state. The end points of the VAS were defined as: 0 = “non-responsive” and 10 = “fully awake”. A score of ≥ 5 was required from both the clinician and the patient for the participant to be randomised into the study. If a patient or clinician reported a VAS < 5 approximately 2 h after surgery, they were excluded from the study. A score of > 5 was chosen based on consensus opinion in this setting as it was intended that the patients would act as their own control for continued participation in the trial based on their own perception of their wakefulness. The clinician’s input would be to moderate this self-assessment based on their clinical experience in managing numerous post-operative women.

Once randomised, the participant’s bed was positioned between 10° and 45°. Following which, patients commenced the 30-min protocol that consisted of two 10-min segments of VRT interspersed with one 10-min washout period. For patients assigned to intervention group 1, this entailed active VRT (10 min), followed by a washout period (10 min) and then passive VRT (10 min). Intervention group 2 underwent passive VRT, followed by a washout period and then active VRT. The washout period consisted of routine clinical care and was intended to allow for the analgesic effects of VRT to dissipate. A 10-min washout has been deemed adequate in experimental studies for the effect of VRT to dissipate^19^.

During the study, participants were welcome to cease VR at any stage of the trial, in which case, they were recorded as a withdrawal and their reason for cessation was also documented.

### VR content

Two pieces of VR content, delivered via the Oculus Go headset, were used during this trial, both of which were developed by ALO VR (Singapore) specifically for this trial. The pieces of content were “Sky Lights 2” and “Cosmic You”, the former being the active distraction content and the latter, the passive meditation content.

**Figure 1:**
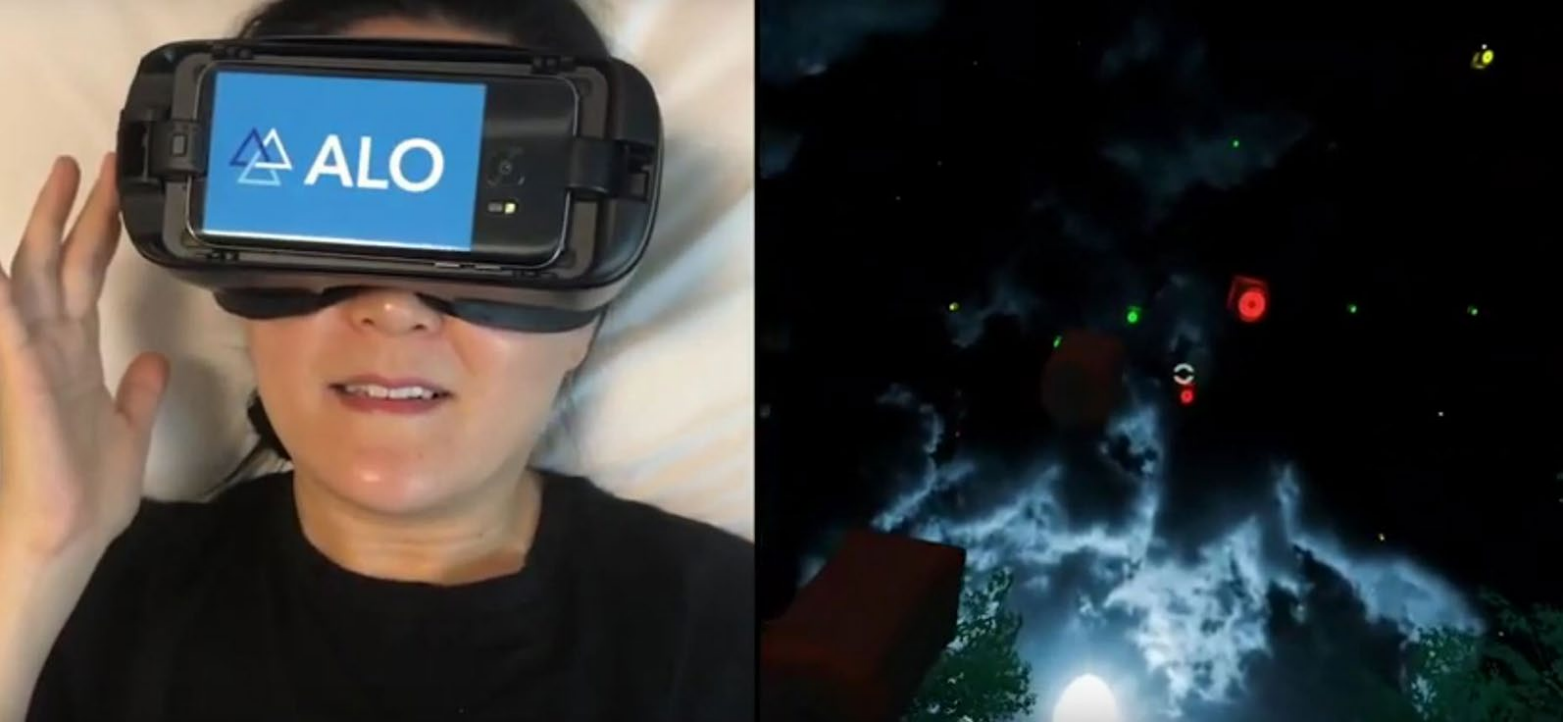
Picture of a user experiencing the Sky Lights 2 immersive pain distraction content.

In Sky Lights 2 (Fig. 1), the user is placed lying down on their back in a quiet field, staring at a starry night sky with several unlit Chinese lanterns floating gently above. By focusing their gaze on a lantern, the user can set it alight, causing the lantern to rise upwards and away. Occasionally, as a reward for continued participation; a lit lantern will either set off a series of fireworks or form Lantern Festival shapes such as a dragon or a giant fish. Relaxing background music is also played to provide auditory stimulation. For this trial, user control was achieved by head tracking and lanterns were lit through triggers on either a Bluetooth hand controller or touchpad on the head-mounted display, based on user preference. Orientation to the device and instructions required approximately 60 s and the procedure itself only commenced once the headset was secured onto the patient and verbal confirmation was received that the game had started.

In Cosmic You, participants are listening to a guided meditation with relaxing background music whilst looking at colourful shooting stars in the night’s sky. Users are not required to interact with this content but are encouraged to participate in the meditation.

The rationale for comparing the analgesic effects of active distraction VR versus passive meditation VR was to determine if there is a relationship between therapeutic effect and the degree of interaction required by the participant. In a systematic review by Smith et al., it was identified that the analgesic effect is significantly greater for active VR than for passive VR, although clinical no studies had explicitly investigated this difference^14^. As such, this study is perhaps one of the first clinical studies to do so.

### Statistical methods

#### Sample size

In order to assess the primary outcome of comparing the differences between the active and passive VR content and their respective effects on pain scores across the various time points, power calculations were prepared using a within subject repeated measures ANOVA model for 2 groups and 4 repeated measurements. The assumptions were an effect size of 0.23, a correlation between repeated measures of 0.5 and a non-sphericity correction of E = 1, β = 95% and α = 0.05^23^. This approximated a sample size of 30. Considering a 10% loss to follow up, 34 women were to be recruited into the study with n = 17 in each treatment arm. It was also established a priori that the trial will continue recruiting until the adequate numbers were available in each arm to also establish the level of drop out with VRT use.

### Data analysis

The raw data for numerical variables in the study were assessed for their distribution using the Shapiro–Wilk test. Normally distributed data were expressed as mean (± standard deviation), whilst skewed data were expressed as median (inter-quartile range). Categorical variables were expressed as frequency counts (percentages).

For the primary outcome, comparing the effects of the active and passive VR contents on pain scores, two-way mixed model ANOVA analysis was employed to investigate differences between the groups. For each parameter, all data points: 0, 10, 20, and 30 min were assessed. The data were primarily assessed for the two-way interaction of treatment and time. If significant, post-hoc analysis with a Bonferroni correction for multiple comparisons was employed. In the event of a non-significant two-way interaction term, subsequent analysis for the effect of time and the effect of treatment were carried out.

For the secondary outcome, comparing the effects of active and passive VR content on opioid requirements, Mann–Whitney U test was used to analyse between group differences. To compare the effects of active distraction and passive meditation VR content on side effects, Fisher’s exact test or the corrected Fisher–Freeman–Halton exact test was employed.

For the remaining variables, the statistical test used to compare the intervention groups was dependant on the data. Continuous variables were analysed for differences either via the independent sample t-test or the Mann–Whitney U test, dependent on their distribution. Categorical variables were compared using either chi-squared test of homogeneity, Fisher’s exact test or the corrected Fisher–Freeman–Halton exact test, dependent on the expected values in the frequency table. In each of aforementioned statistical tests, the null hypothesis was defined as no difference between the intervention groups’ mean, median or frequency.

Unless otherwise specified, the assumptions of all statistical tests were met. For all tests, statistical significance was set at an alpha level of 0.05.

Statistical analysis was completed on SPSS v24.0 and figures were created using GraphPad v7.0b.

## Results

### Patient characteristics

**Figure 2:**
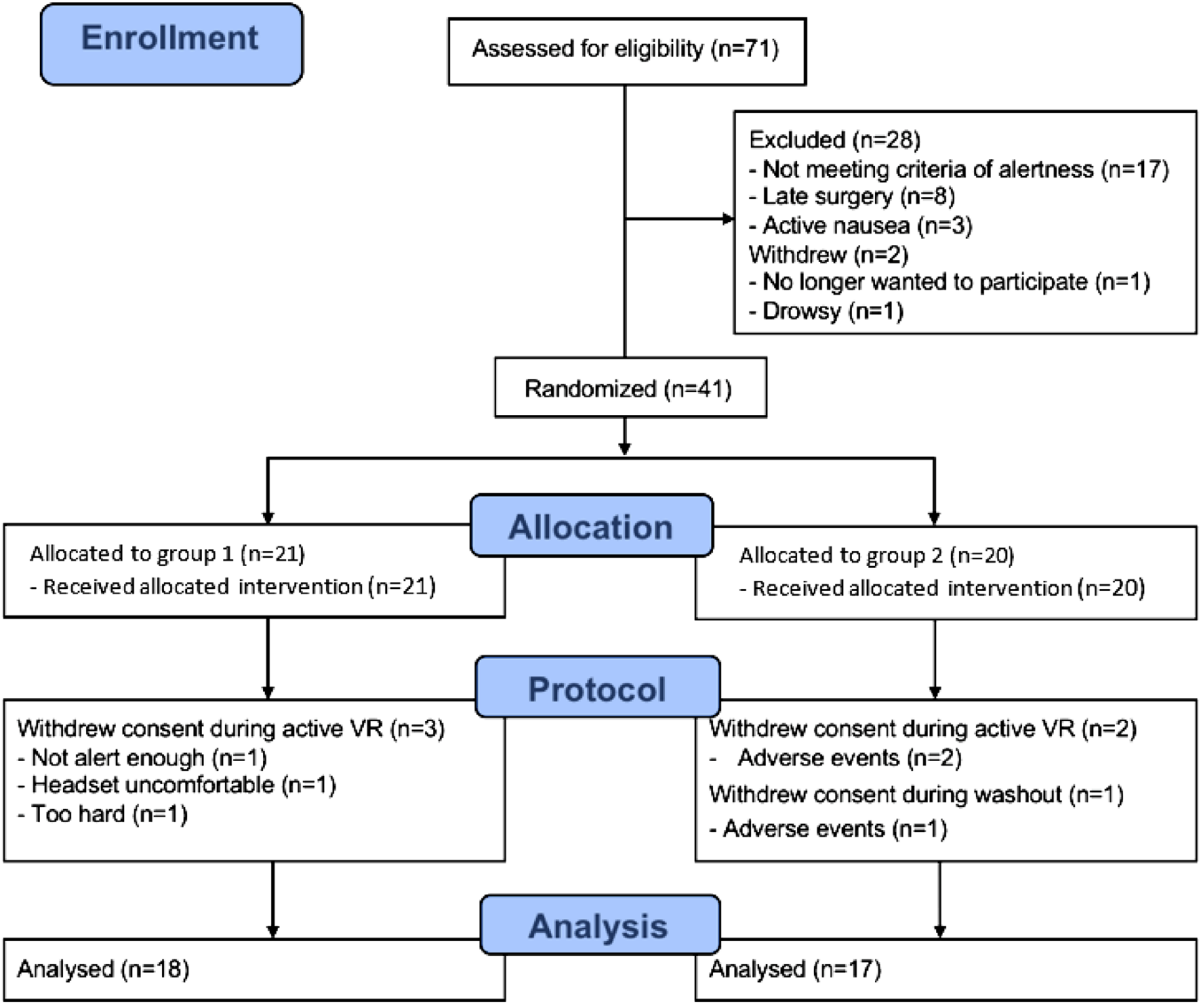
CONSORT flow diagram.

35 women completed the study (Fig. 2). 18 women were randomised into intervention group 1, and 17 women into the intervention group 2. The baseline patient characteristics are presented in Table 1. There were no significant differences in baseline characteristics between the intervention groups.

**Table 1:** Baseline patient characteristics. Mean (± standard deviation), Median (25th percentile to 75th percentile), ^+^Independent samples t-test, ^⊗^Mann–Whitney U-test.

Parameters	Results for Group 1 (n = 18)	Results for Group 2 (n = 17)	p-value
Age (years)	39.27 (± 10.20)	41.67 (± 12.92)	0.54^+^
BMI (kg/m^2^)	30.61 (± 7.85)	30.19 (± 9.03)	0.84^+^
Duration of Surgery (minutes)	69.50 (47.50–105.75)	61.00 (51.00–76.50)	0.46^⊗^
Wakefulness of patient-PACU clinician rated (11-point scale)	7.00 (5.00–8.00)	7.00 (6.00–8.00)	0.76^⊗^
Wakefulness of patient—patient rated (11-point scale)	5.00 (5.00–7.00)	6.00 (5.00–7.50)	0.59^⊗^

### Pre-intervention results

The responses to the pre-procedural questionnaire are presented in the Supplementary materials Table A. There were no significant differences between the groups.

### Primary outcomes

#### Pain scores

**Figure 3:**
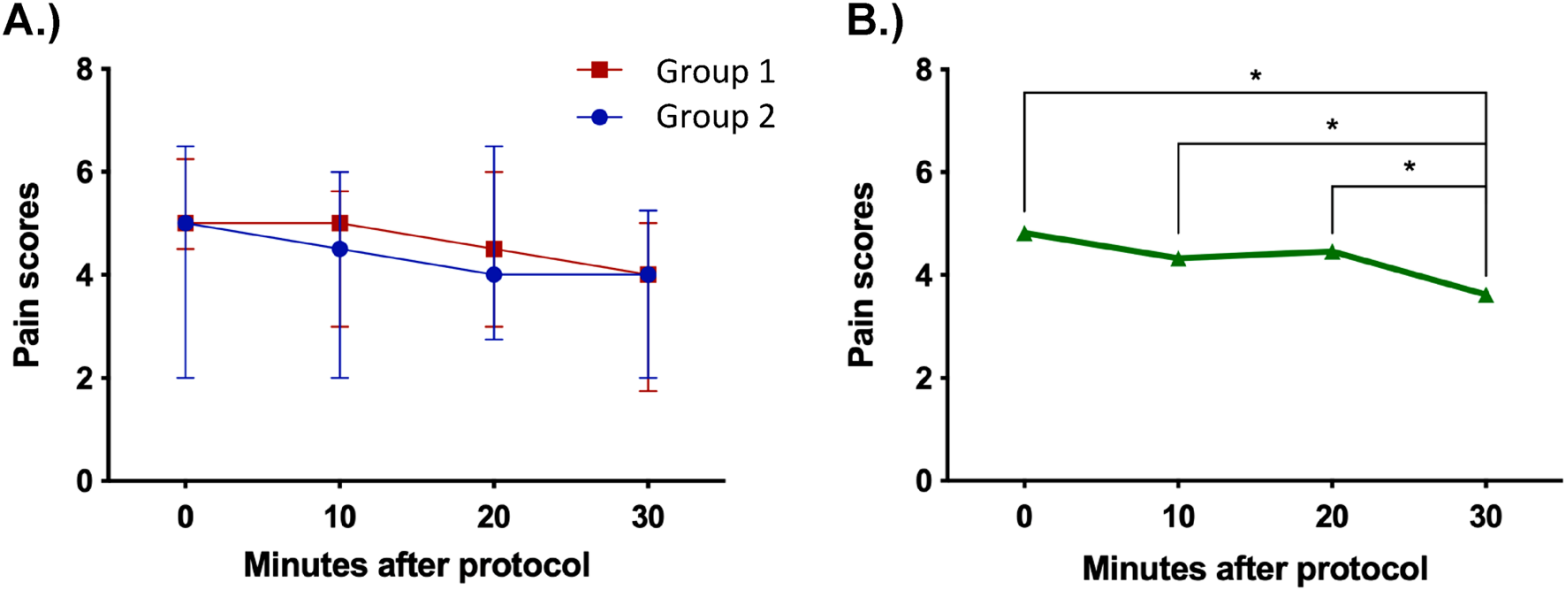
(**A**) Median pain scores with interquartile range for intervention group 1 (n = 18) and intervention group 2 (n = 17) (Supplementary material) (**B**) Mean pain scores for the entire sample size (n = 35) at 0, 10, 20 and 30 min. The first segment of VR was administered between 0 and 10 min and the second segment between 20 and 30 min. Intervention group 2 were administered passive then active content, whilst intervention group 1 were administered active then passive content. A two-way mixed model ANOVA with multiple comparisons and a Bonferroni correction was used to determine significance. *Denotes p < 0.05.

The main effect of time showed a statistically significant difference in mean pain scores across the different time points [F(2.12, 70.01) = 8.63, p < 0.0005, partial η^2^ = 0.21]. The main effect of treatment showed that there was no statistically significant difference in pain scores between treatment groups [F(1, 33) = 0.23, p = 0.64, partial η^2^ = 0.007]. There was no statistically significant interaction between treatment (group 1/group 2) and time on pain scores [F(2.12, 70.01) = 2.71, p = 0.071, partial η^2^ = 0.076]. Post-hoc analysis with a Bonferroni adjustment of the main effect of time revealed that pain scores significantly reduced from 0 to 30 min (1.18 [95% CI 0.31–2.05], p = 0.003), from 10 to 30 min (0.69 [95% CI 0.034–1.35], p = 0.035) and from 20 to 30 min (0.82 [95% CI 0.24–1.41], p = 0.002) (Fig. 3). The remaining three pairwise comparisons showed no significant difference (p > 0.05).

### Feasibility and acceptability

In this study, we had a recruitment rate of 57.7% (41/71). Of the thirty patients who were excluded or withdrew prior to randomisation, 56.7% (17/30) were as a result of the patient not meeting the criteria for alertness within two hours of surgery. Overall, 14.6% (6/41) of patients dropped out of the trial following randomisation. Of those withdrawals, 83.3% (5/6) did so during the administration of active VRT, whilst 50.0% (3/6) were in relation to adverse side effects (nausea) encountered during VRT.

The responses to the post-intervention questionnaire are presented in Supplementary materials Table C. Intervention group 1 rated the experience of using the VR device significantly better than intervention group 2 [mean ± SD = 7.97 (± 1.22) vs. 6.62 (± 1.92), p = 0.017]. No other significant differences in responses were observed between treatment groups. Overall, the vast majority of participants would use VRT as the standard of care in their next procedure (97.1%, n = 34) and recommend the device to a friend (94.3%, n = 33).

### Secondary outcomes

#### Opioid use

A total of 20 (57.1%) patients were administered opioids post-operatively. The opioid equivalents administered across the trial are illustrated in Table 2. Pre-protocol, intervention group 1 were administered a significantly greater number of opioid equivalents prior to the commencement of protocol than intervention group 2 [18.0 (14.5–27.0) vs. 6.0 (0.0–12.0), p = 0.01]. However, between 0 and 10 min, intervention group 1 were administered a significantly lower number of opioids compared to intervention group 2 [0.0 (0.0–7.5) vs. 3.0 (0.0–10.0), p = 0.04]. Additionally, a smaller proportion of intervention group 1 received PO opioids in comparison to intervention group 2 (9/18 compared to 11/17). Only three participants in the intervention group 1 received opioids during protocol compared to nine participants in intervention group 2 (Supplementary materials Table D).

**Table 2:** Median (IQR) opioid equivalents administered to the intervention groups: prior to commencing protocol; between 0–10; 10–20; and 20–30 min. Mann–Whitney U-test, *Denotes significance.

Opioid requirements	Intervention group 1	Intervention group 2	p-value
Pre-protocol	18.0 (14.5–27.0)	6.0 (0.0–12.0)	0.01*
0–10 min	0.0 (0.0–7.5)	3.0 (0.0–10.0)	0.04*
Washout at 10–20 min	0.0 (0.0–0.0)	0.0 (0.0–7.5)	0.30
20–30 min	0.0 (0.0–0.0)	0.0 (0.0–5.0)	0.77
Total (pre-protocol to 30 min)	6.0 (0.0–24.4)	12.0 (0.0–19.5)	0.86

### Side effects

Table 3 presents the side effect results. At 30 min, intervention group 2 experienced significantly more side effects than intervention group 1 [41.2% (n = 7) vs. 5.6% (n = 1), respectively, p = 0.014]. However, no other significant differences were observed between groups.

**Table 3:** The side effects (SE) reported by participants at 0, 10, 20 and 30 min and the total number of participants that experienced side effects. ^a^Fisher’s exact test, ^b^Fisher–Freeman–Halton exact test. ^c^Denotes significance.

	Results for intervention group 1 (n = 18)	Results for intervention group 2 (n = 17)	p-value
**SE at 0 min**			
Nausea and/or dizziness	1 (5.6%)	1 (5.9%)	1.00^a^
No SE	17 (94.4%)	16 (94.1%)	
**SE at 10 min**			
Nausea and/or dizziness	2 (11.1%)	2 (11.8%)	1.00^b^
No SE	16 (88.9%)	15 (88.2%)	
**SE at 20 min**			
Nausea and/or dizziness	2 (11.1%)	2 (11.8%)	1.00^b^
No SE	16 (88.9%)	15 (88.2%)	
**SE at 30 min**			
Nausea and/or dizziness	1 (5.6%)	7 (41.2%)	0.014^b,c^
No SE	17 (94.4%)	10 (58.8%)	
**Overall SE**			
Experienced SE	2 (11.1%)	7 (41.2%)	0.06^a^
No SE	16 (88.9%)	10 (58.8%)	

## Discussion

This pilot study provides valuable information regarding the utilisation of VR therapy as an analgesic following laparoscopic surgery for POP. Furthermore, based on our a priori criteria for success, we consider the following pilot to be successful and worthy of formal evaluation through a clinical trial.

### Primary outcomes

#### Pain scores

The trial demonstrated a significant reduction in pain over time. Although there is a paucity of data, the available evidence suggests that in similar populations post-laparoscopy, pain tends to increase in the first two hours following surgery, even with the use of opioid analgesia^24–27^. Furthermore, although non-significant, it is of interest that for the entire sample regardless of intervention group that pain scores increased during the washout period (10–20 min). These findings do raise some suggestion around whether VRT may be exerting some analgesic effect. These therapeutic effects of VRT appear to be corroborated by the available literature, where it is proposed that VRT induces its effect through distraction and neurophysiological mechanisms^6,9,14^. However, in our study, there appears to be no discernible differences between active distraction and passive meditation VRT in the analgesic effect conferred to participants. Considering the scarcity of available data, the available literature on this matter is equivocal at best. When relying on available clinical and experimental studies, these inconsistent findings are potentially related to the indication for which VR has been applied within the clinical subpopulations being investigated^15,18,28^.

### Feasibility and acceptability

Despite the large number of participants who were excluded from the study prior to randomisation due to their lack of alertness (n = 17), we had a markedly better rate of retention post-randomisation. A total of 6 participants (14.6%) withdrew during protocol, which does not drastically differ from our expected loss to follow up of 10%. It deserves consideration however, that the majority of these women withdrew whilst receiving active VRT. This may be related to several of reasons such as (1) poor familiarity with the use of the interactive elements of the technology, (2) worsening of post-operative side effects due to the discrepancy which occurs between the ocular and vestibular systems when the senses do not receive the usual sensory feedback that would be expected in such a scenario, or (3) lack of satisfaction with the active content^14^. This will be an element of future investigation. Furthermore, our pilot demonstrated overall high rates of acceptance of VRT in a POP setting, indicating that the device is well received as an adjunct analgesic, which would aid future implementation.

### Secondary outcomes

#### Opioid use

The opioid requirements of intervention group 1 were significantly greater than the intervention group 2 prior to VRT administration. However, between 0 and 10 min when intervention group 1 were administered active VR, their opioid requirements became significantly less than intervention group 2 who were administered passive VR during this time frame. Although this effect is not observed between 20 and 30 min when the allocation of VR content was reversed, the opioid requirements by this stage are low in both groups [0.0 (0.0–0.0) vs 0.0 (0.0–5.0)]. This may be an important finding since it is in contrast to the available literature that suggests a consistent increase in opioid requirements in the acute PO period following gynaecological laparoscopy^29^.

In elucidating the potential mechanism behind this effect, the literature has demonstrated, via functional MRI (fMRI), how active VRT can work synergistically as an adjunct analgesic to further reduce opioid requirement in patients^30,31^. Furthermore, this effect appears to act in a dose-dependent fashion, based on the quality and degree of distraction^14,19^. This potentially suggests that active VRT has the potential to function better than passive VRT, which could explain the significant difference in opioid requirements between 0 and 10 min. Cumulatively, these findings do raise the potential of active distraction VR being effective within the PO setting in reducing opioid analgesia requirements. In saying this however, there is a realisation that this should be rigorously investigated within the context of a well-designed RCT since this effect could potentially be the result of the intervention group receiving more opioids initially.

### Side effects

Our pilot study revealed that 25.7% (n = 9) of patients experienced side effects, which did not significantly differ between the groups. Again, this was below the level of our a priori criteria of 30% to consider the pilot a success and safe.

Additionally, it is important to note that 5.7% (n = 2) of the women experienced dizziness prior to VRT. In contrast to these results, current evidence suggests that the incidence of side effects due to VRT is between 0 and 6%, a much lower rate than we encountered^15,32–39^. However, these studies are not comparable to our own as they were not undertaken post-operatively, where exposure to general anaesthetics can induce similar side effects to VRT^40,41^. The incidence of PO nausea and vomiting (PONV) following a gynaecological laparoscopy is between 20 and 37% in the first two PO hours^39^. Despite the fact that PONV was not the only SE that we observed, it was the most prevalent, potentially explaining why we encountered such high rates of SE in participants compared to other VR studies.

The only significant difference in SE occurred at 30 min. At this time point intervention group 2 experienced significantly more SE than intervention group 1 (p = 0.0014) after being administered the active VR, mainly due to an increased incidence of nausea (29.4% vs. 0%). It begs consideration however, that at 10 min, when intervention group 1 had completed their active VR segment, no patients experienced nausea. This suggests that it may be either be VRT in general, rather than content type, or the SE experienced routinely within the PO period which may have been contributory towards these findings.

### Limitations

This study has several limitations that should be considered when interpreting its results. Primarily, the first is in relation to the small sample size being utilised. However, as the study was conducted as a proof of concept to determine the feasibility, acceptability and adverse outcomes of VR, the results are still valuable for aiding the development of future trials. Additionally, the sample size was adequately powered for our primary objective of comparison between active and passive VR. In terms of statistical methodology, there was also some deliberation around the use of the ANOVA versus the paired t-test in comparing pain scores for this study. However, considering the potential of carry over and/or period effects in affecting pain scores and sample size (n > 30), it was deemed by the statistical team that ANOVA was the more appropriate test.

Also, the lack of a ‘no distraction’ control group proved to be a major limitation to our study. The inclusion of such a group would not only have allowed us to further determine the effect of VR on pain scores but also additionally would have allowed us to quantify the prevalence of side effects during the post-operative periods in patients undergoing routine care. Furthermore, there was a lack of thorough evaluation around the reasons behind the reasons for dropping out in 2 patients which would have been helpful in defining contributory factors of real-world non-compliance to treatment.

To address these factors, our future study will encompass an RCT design with a control group undergoing routine care in comparison to active VRT to allow rigorous comparison and address the following factors. Furthermore, we will carry out qualitative testing of patients dropping out to better define their reasons for not continuing with therapy. This will also include considerations on controlling for the wakefulness of the patient as a potential confounding variable for pain, which was not investigated in this trial.

The next limitation is the lack of an evaluation of participant immersion in the VR experience, which was not performed in this study. Given the development of new VR worlds and technologically advanced headsets, there is potential that the immersiveness of the VR experience has increased, which could provide greater therapeutic benefit than that seen in this study and the literature to date. As such, our future research will also endeavour to include immersion as an outcome measure.

Finally, a better comparison of active versus passive VRT could have been achieved if the contents were the same, with the passive content modified to restrict user inputs in the interactive component so that the user is simply watching the content. This will be a consideration for our future research into this aspect of VRT as a non-pharmacologic analgesic.

## Conclusions

This pilot study demonstrated that VRT has the potential to be utilised as an analgesic modality for POP in a safe manner. However, it did not demonstrate any substantial differences in analgesic effect between active distraction and passive meditation VRT. Instead, it raised some suggestions as to the potential to modulate opioid requirements during the PO period. Importantly, the study demonstrated high levels of patient experience and acceptability associated with VRT use. This sets the foundations for further rigorous evaluation of VRT against routine care in the manner of a formalised RCT.

## Supplementary Information

Supplementary Information.

## Data Availability

The datasets generated during and/or analysed during the current study are not publicly available to protect individual patient information and data but are available from the corresponding author on reasonable request.

## References

[1] Gerbershagen HJ (2013). Pain intensity on the first day after surgery: A prospective cohort study comparing 179 surgical procedures. Anesthesiology.

[2] Jarrell J (2014). Prediction of postoperative pain after gynecologic laparoscopy for nonacute pelvic pain. Am. J. Obstet. Gynecol..

[3] Wheatley SA, Millar JM, Jadad AR (1994). Reduction of pain after laparoscopic sterilisation with local bupivacaine: A randomised, parallel, double-blind trial. BJOG Int. J. Obstet. Gynaecol..

[4] Ekstein P (2006). Laparoscopic surgery may be associated with severe pain and high analgesia requirements in the immediate postoperative period. Ann. Surg..

[5] *Medical Devices and Surgical Technology Week; Atlanta* (NewsRx, 2016).

[6] Mallari B, Spaeth EK, Goh H, Boyd BS (2019). Virtual reality as an analgesic for acute and chronic pain in adults: A systematic review and meta-analysis. J. Pain Res..

[7] Enright A, Goucke R (2016). The global burden of pain: The tip of the iceberg?. Anesth. Analg..

[8] Kehlet H, Jensen TS, Woolf CJ (2006). Persistent postsurgical pain: Risk factors and prevention. Lancet.

[9] Gupta A, Scott K, Dukewich M (2018). Innovative technology using virtual reality in the treatment of pain: Does it reduce pain via distraction, or is there more to it?. Pain Med..

[10] Trost Z (2015). The promise and challenge of virtual gaming technologies for chronic pain: The case of graded exposure for low back pain. Pain Manag..

[11] Hoffman HG, Doctor JN, Patterson DR, Carrougher GJ, Furness TA (2000). Virtual reality as an adjunctive pain control during burn wound care in adolescent patients. Pain.

[12] Wiederhold BK, Soomro A, Riva G, Wiederhold MD (2014). Future directions: Advances and implications of virtual environments designed for pain management. Cyberpsychol. Behav. Soc. Netw..

[13] Ding L (2020). Effects of virtual reality on relieving postoperative pain in surgical patients: A systematic review and meta-analysis. Int. J. Surg..

[14] Smith V (2020). The effectiveness of virtual reality in managing acute pain and anxiety for medical inpatients: Systematic review. J. Med. Internet Res..

[15] Tashjian VC (2017). Virtual reality for management of pain in hospitalized patients: Results of a controlled trial. JMIR Ment. Health.

[16] Chan E, Foster S, Sambell R, Leong P (2018). Clinical efficacy of virtual reality for acute procedural pain management: A systematic review and meta-analysis. PLoS One.

[17] Smith V (2020). A randomised controlled trial to assess the feasibility of utilising virtual reality to facilitate analgesia during external cephalic version. Sci. Rep..

[18] Phelan I (2019). A mixed-methods investigation into the acceptability, usability, and perceived effectiveness of active and passive virtual reality scenarios in managing pain under experimental conditions. J. Burn Care Res..

[19] Lier EJ, Oosterman JM, Assmann R, de Vries M, van Goor H (2020). The effect of Virtual Reality on evoked potentials following painful electrical stimuli and subjective pain. Sci. Rep..

[20] Williamson A, Hoggart B (2005). Pain: A review of three commonly used pain rating scales. J. Clin. Nurs..

[21] Hjermstad MJ (2011). Studies comparing numerical rating scales, verbal rating scales, and visual analogue scales for assessment of pain intensity in adults: A systematic literature review. J. Pain Symptom Manag..

[22] Faculty of Pain Medicine ANZCA. *Opioid Dose Equivalence*. https://fpm.anzca.edu.au/documents/opioid-dose-equivalence.pdf (2019).

[23] Whitehead AL, Julious SA, Cooper CL, Campbell MJ (2016). Estimating the sample size for a pilot randomised trial to minimise the overall trial sample size for the external pilot and main trial for a continuous outcome variable. Stat. Methods Med. Res..

[24] Li M (2012). Propofol reduces early post-operative pain after gynecological laparoscopy. Acta Anaesthesiol. Scand..

[25] Abdelazim IA, Al-Kadi M, Shourbagy MME, Mohamed AA, Faza MLA (2013). Intraperitoneal lidocaine and tenoxicam for pain relief after gynaecological laparoscopy. Asian Pac. J. Reprod..

[26] Baradwan S (2022). Preoperative duloxetine on postoperative pain after laparoscopic gynecological surgeries: A systematic review and meta-analysis of randomized controlled trials. J. Gynecol. Obstet. Hum. Reprod..

[27] Hatami M, Kheirati A, Behdad S, Javaheri A, Vaziribozorg S (2020). The effect of preoperative administration of duloxetine on postoperative pain after laparoscopic myomectomy. Acta Med. Iran..

[28] Piskorz JE, Czub M, Šulžickaja B, Kiliś-Pstrusińska K (2020). Mobile virtual reality distraction reduces needle pain and stress in children?. Cyberpsychol. J. Psychosoc. Res. Cyberspace..

[29] Lenz H, Sandvik L, Qvigstad E, Bjerkelund CE, Raeder J (2009). A comparison of intravenous oxycodone and intravenous morphine in patient-controlled postoperative analgesia after laparoscopic hysterectomy. Anesth. Analg..

[30] Hoffman HG (2006). Using FMRI to study the neural correlates of virtual reality analgesia. CNS Spectr..

[31] Hoffman HG (2007). The analgesic effects of opioids and immersive virtual reality distraction: Evidence from subjective and functional brain imaging assessments. Anesth. Analg..

[32] Hoffman HG (2008). Virtual reality pain control during burn wound debridement in the hydrotank. Clin. J. Pain.

[33] Walker MR (2014). Treatment efficacy of virtual reality distraction in the reduction of pain and anxiety during cystoscopy. Mil. Med..

[34] Chan EA, Chung JW, Wong TK, Lien AS, Yang JY (2007). Application of a virtual reality prototype for pain relief of pediatric burn in Taiwan. J. Clin. Nurs..

[35] Frey DP (2018). Virtual reality analgesia in labor: The VRAIL pilot study-a preliminary randomized controlled trial suggesting benefit of immersive virtual reality analgesia in unmedicated laboring women. Anesth. Analg..

[36] Birnie KA (2018). Usability testing of an interactive virtual reality distraction intervention to reduce procedural pain in children and adolescents with cancer [Formula: see text]. J. Pediatr. Oncol. Nurs..

[37] Gold JI, Mahrer NE (2017). Is virtual reality ready for prime time in the medical space? A randomized control trial of pediatric virtual reality for acute procedural pain management. J. Pediatr. Psychol..

[38] Mosso-Vazquez JL, Gao K, Wiederhold BK, Wiederhold MD (2014). Virtual reality for pain management in cardiac surgery. Cyberpsychol. Behav. Soc. Netw..

[39] Alshatrat SM, Alotaibi R, Sirois M, Malkawi Z (2018). The use of immersive virtual reality for pain control during periodontal scaling and root planing procedures in dental hygiene clinic. Int. J. Dent. Hyg..

[40] Benyamin R (2008). Opioid complications and side effects. Pain Physician.

[41] Purhonen S, Koski EM, Niskanen M, Hynynen M (2006). Efficacy and costs of 3 anesthetic regimens in the prevention of postoperative nausea and vomiting. J. Clin. Anesth..

