# Supplementary material for: Virtual reality and its use in post-operative pain following laparoscopy: a feasibility study
Source: Sci Rep. 2022 Jul 30;12:13137. doi: 10.1038/s41598-022-17183-2 (PMC9338945; doi:10.1038/s41598-022-17183-2)
Supplement: Supplementary file 1 — Supplementary Information. [file 41598_2022_17183_MOESM1_ESM.docx]

**Supplementary Materials: Study protocol, pre-operative questionnaire, post-intervention questionnaire, tables A, B, C and D.**

**Title: Virtual reality and its use in post operative pain following Laparoscopy - a feasibility study.**

**Authors:** Olivia Payne^1#^, Vinayak Smith^1,3#^*, Daniel L. Rolnik^1^, Miranda Davies-Tuck^1,4^, Ritesh Warty^1^, Densearn Seo^5^, Lima Wetherell^1^, Hamsaveni Kalina Murday^1^, Amrish Nair^3^, Rashvinder Kaur^3^, Beverley Vollenhoven^1,2^

**Study Protocol**

Study Title: A clinical pilot to investigate the effect of virtual reality on post laparoscopy pain scores

Short Title: Virtual reality analgesia for post laparoscopic pain

Study Investigator(s):

Principal Investigator (A): Prof Beverley Vollenhoven ^1^

Phone: +61335345145

Email: Beverley.vollenhoven@monash.edu

Principal Investigator (B): Dr Vinayak Smith ^1^

Phone: +61431330754

Email: vinayak.smith@monash.edu

Associate Investigator (A):

Phone:

Email:

Associate Investigator (B):

Phone:

Email:

**Institution**

^1^ Department of Obstetrics and Gynaecology, Monash University, Clayton, VIC 3800

**1. INTRODUCTION**

The following study will be a comparative open label randomised human pilot study for patients utilising a novel therapeutic modality technology- virtual reality (VR) - to assess its utility as an analgesic in acute pain post laparoscopy. The following pilot study is aimed at efficacy and will assess:

- The effect of VR on pain scores after laparoscopy
- The effect of VR on physiological parameters after laparoscopy
- The effect of VR on opiate analgesia requirements after laparoscopy
- The magnitude of side effects generated by VR
- Patient feedback on device experience

This will be achieved by comparing VR to routine care post laparoscopy.

As such, the following study will aid in formulating evidence for utilising VR technology as an analgesia option post laparoscopy and potentially lay the foundations for a larger scale randomised controlled trial (RCT).

**2. BACKGROUND**

Post-operative pain following surgery remains an area of concern for patients and clinicians alike and can affect between 20-40% of patients. Available evidence shows that even in the context of modern pain management strategies, it is an area which causes significant morbidity to many patients and is a parameter which frequently underestimated by clinicians as well^1 2^. The immediate concerns relating to poor analgesia control in these patients include decreased patient satisfaction, delayed postoperative ambulation, delayed discharge from the hospital and the propensity for the development of chronic pain syndromes ^1 3^.

Laparoscopy is a commonly performed gynaecological procedure. The studies surrounding post-operative pain in these women are sparse and conflicting. They however suggest the frequency of post-operative pain to be between 35 – 65%^4^. The primary mechanisms of pain are theorised to be related to diaphragmatic irritation and peritoneal inflammation and stretching ^5^. Ekstein et al. demonstrated that in the postoperative period ( 4 hours post-surgery) , nearly 46% of patients post laparoscopy were in severe pain [≥ 6 on the visual analogue scale (VAS)] and they required significantly greater amounts of analgesia in comparison to patients undergoing laparotomy^6^. Gerbershagen et al. further outlined the underestimation and reduced treatment of pain in these patients as well by demonstrating that patients with high post laparoscopy pain scores were administered lower level of opioid analgesia in comparison to other surgical procedures with equivalent pain scores^1^.

Virtual reality (VR) is a technological medium that is used to create simulated scenarios in which users are immersed and able to interact with the virtual environment (VE) through multisensorial stimulation^7^. There has been a recent interest in assessing its utility to provide analgesia in various field of medicine. As it stands, VR has demonstrated clinical efficacy in pain reduction whilst being well tolerated by patients as well ^8-10^. Importantly, a recent controlled trial by Tashijan et al. demonstrated a 24% drop in pain scores in patients utilising VR for acute pain post operatively ^11^. At present, there are several theories behind its mechanism of action on how VR facilitates analgesia. Although the primary mechanism is unknown, it is theorised that it entails a mixture of distraction to the user as well as the evoking of neurophysiological changes^10^.

Given the potential of VR to facilitate analgesia, the question of whether it can make post laparoscopy pain less painful and reduce opiate dependence does arise. To date, its utility post laparoscopy in particular is unknown and no trials have been conducted to address this. Decreased post-operative pain has the positives of increased rates of patient satisfaction and decreased requirement for opiate analgesia^12^. Importantly, for day case laparoscopies, it does give the advantage of immediate discharge from the hospital thereby enacting cost saving benefits to the healthcare system as well

As such, there remains a significant clinical gap in judging the utility of VR in providing analgesia post-operatively. This prompted us to design a pilot study to evaluate the efficacy of VR in this context. It is hoped that this can be used as a platform to design a larger sized controlled study should the results appear promising.

**3. AIM(S) AND OBJECTIVES OF STUDY**

The aims and objectives of the following study is to function as a proof of concept study for examining the effect of VR on:

- Pain scores post laparoscopy
- Physiological parameters post laparoscopy
- Analgesia requirements post laparoscopy
- Same day discharge rates post laparoscopy
- Side effects generated and acceptability in participants
- Patients feedback of the device

**4. HYPOTHESIS**

**Primary Hypothesis**

H_0_: VR has no effect on pain scores post laparoscopy

H_A_: VR has an effect on pain scores post laparoscopy

**Secondary Hypothesis**

H_0_: VR has no effect on side effects post laparoscopy

H_A_: VR has an effect on side effects post laparoscopy

**5. STUDY DESIGN**

The following study is a clinical crossover pilot study with randomisation of treatment. The following design has been chosen as it is considered to be the design of choice in evaluating a diagnostic test^13^.

**6. STUDY SETTING/LOCATION**

The following study will be carried out at Moorabbin Day Surgery Centre. The following study is a single centred study.

**7. STUDY POPULATION**

The study population will be recruited from patients referred in to Day Surgery who meet the inclusion criteria. For the analysis, 50 women will be recruited via convenience sampling for the proof of concept.

**8. ELIGIBILITY CRITERIA**

**Inclusion criteria**

The women in the following group:

1. Undergoing laparoscopy for any indication

The following criteria are relevant as:

1. All indication for gynaecological laparoscopy were considered suitable for pain assessment

**Exclusion criteria**

1. Conversion to laparotomy
2. Chronic narcotic use / dependence
3. Pregnancy
4. Prior history of sensitivity to VR technology, motion sickness, vertigo, seizures, epilepsy and active nausea and vomiting
5. Women below 18 years of age
6. Patients with an intellectual or mental impairment
7. People in existing dependent or unequal relationships with any member of the research team, the researcher(s) and/or the person undertaking the recruitment/consent process
8. People highly dependent on medical care

**9. STUDY OUTCOMES**

**Primary Outcome**

The primary outcomes for the following study will include:

1. Correlation between VR utilisation and pain scores
2. Correlation between VR utilisation and changes in physiological parameters
3. Correlation between VR and amount of post-operative opiate analgesia used
4. Correlation between VR and side effects experienced by participants

**Secondary Outcome**

1. Demographic data of participants who were recruited
2. Patient feedback on device experience

**10. STUDY PROCEDURES**

**a. Participant recruitment**

Participants will be recruited via convenience sampling from day surgery unit. The potential for spectrum bias to be introduced into the study due to this method of sampling is present. However, for a proof of concept phase and to reduce patient inconvenience, the following sampling framework has been chosen.

**b. Randomisation and Blinding**

For the following study, randomisation for passive or active content will be carried out via randomised permutated blocks using Microsoft Excel 2016. No blinding will be carried out for the analysis and interpretation of the results as well.

**c. Study procedures**

Patients will be approached prior to their surgery in the day procedure unit. Patient will initially be screened as per the inclusion and exclusion criteria and pre-consent will be obtained. Patients will be counselled at this juncture that their inclusion is also dependent on their post-operative clinical state as well. Prior to the monitoring, the women will be asked a few questions to obtain their demographic data (Patient questionnaire v 2.0). This will be followed by a measure of their routine observations as well (heart rate, HR: blood pressure, BP: and respiratory rate, RR) This stage will take approximately 10 minutes in duration.

The laparoscopy will be carried out by a surgeon who is experienced (>100 procedures/ surgeon) in the procedure. The surgical technique will be at the discretion of the operating surgeon. The anaesthetic care will be provided by the anaesthetic team at their discretion.

The patients will receive the intervention/ control in the post-acute care area (PACU). The PACU clinician caring for the patient will use a visual analogue scale (VAS) to define the cognitive scale of the patient. In addition, the patient themselves will use a VAS to rate their level of wakefulness. A score ≥ 5 will be required for the patient to be included for the study. The investigator will then randomise the patient to receive either active or passive content first based on the randomised permutated blocks. If the patient has a VAS < 5 they are to be excluded from the study.

**VR Administration**

For the procedure, the woman will be between **10 degrees to** **45-degree to** allow the VR headset to function in the PACU. Patients will fill in a questionnaire regarding their ongoing pain and be evaluated for a range of side effects prior to the VR headset being mounted. Patients will then have the VR headset fitted for them. They will have the immersive VR content, either passive or active, played for them for 10 minutes. Following this, their pain scores will be recorded 2 minutes after 10 minutes of VR. They will then have a 10-minute wash out period of no distraction, following which, their pain scores will be recorded 2 minutes afterwards. They will then be administered the remaining VR content for 10 minutes in the same manner as the first segment. Should the patient request to cease VR at any stage, this will be done and the reason for cessation will be recorded as well.

At each 10-minute juncture, patients will have their side effects and vital signs (HR, BP and RR) recorded.

Post procedure, participants will be administered a questionnaire recording the device experience and feedback on the device as well at the end of the study.

The duration of data collection per patient will not be longer than 60 minutes in total.

For each of the stages, all patient output data will be linked to a single numerical identifier which will be utilised from that point for analysis by the researchers. Data obtained will be securely stored on file on a secure hard disk drive and subsequently uploaded to a cloud server with HIPAA based security.

**d. Measurement tools utilised**

In assessing the primary outcomes:

1. The Numerical rating scale will be utilised to assess pain perception in the following study^14^
   1. This will be measured on a 100mm scale to the closest millimetre (mm)
2. Vital signs will be measured utilising routine BP machines
3. Questionnaires will be used to measure analgesia utilised and side effects encountered. All opiate analgesia will be converted to morphine sulphate equivalents utilising a validated equi analgesic table (Faculty of Pain Medicine, ANZCA)

In assessing the secondary outcomes:

To assess the secondary outcomes, data will be collected directly from the patient through questionnaires.

**e. Safety considerations/ Patient safety**

For the virtual reality headset and content, the technology being utilised is conventional and is available for general consumer use. It has also been previously utilised safely in an in hospital scenario with no documented safety concerns in various settings as well (refer to attached Product Information Guide).

In monitoring the adverse effects associated with utilising the VR headset, screening will be undertaken post intervention through a questionnaire. In addition, the patient information sheet will have the contact of the trial coordinator who they can contact if they experience any safety issues or concerns regarding how the data collection process had been carried out. This information will be returned to the research supervisors who will address the concerns in direct consultation with the patient or advise suitable recourse if the issue is deemed to be under the purview of Monash Health directly.

**11. STATISTICAL CONSIDERATIONS AND DATA ANALYSIS**

**a. Sample size and statistical power**

For the following study, a sample size of 50 was estimated. To detect a mean difference of 1 with a standard deviation of the mean difference of 1.8492 for pain scores, a crossover design study with a power of 0.95 and an alpha of 0.05 requires a sample of 45. Factoring a 10% dropout rate, we aim to recruit 50 women for this study.

**b.** **Statistical methods**

For this study, data will be summarised utilising descriptive statistics.

The normality of the data will then be explored utilising the Shapiro - Wilk and Kolmogorov- Smirnov test.

Within group differences will be compared using the paired t-test. Between group differences (for pain scores, physiological parameters and opiate administration) will be calculated utilising either the unpaired t- test or Wilcoxson signed rank test depending on the distribution of the data. Chi squared tests will be utilised to compare the number of requests for analgesia during the procedure while utilising and not utilising VR.

Regression analysis (univariate and or multivariate) will then be utilised to explore the relationship between VR (passive VR vs. active VR vs. no VR), pain scores and physiological parameters.

The significance level for this study is set at p<0.05.

**12. ETHICAL CONSIDERATIONS**

The study will be conducted in full conformance with principles of the “National Statement on Ethical Conduct in human research”, Good Clinical Practice (GCP) and within the laws and regulations of Australia.

A detailed overview of the ethical considerations is detailed in the attached National Ethics Application Form

**13. OUTCOMES AND SIGNIFICANCE**

Through conducting the following study, we hope to be able to achieve the objective of investigating the efficacy of VR in providing analgesia for patients post laparoscopy. This is the first time VR will be tested as a modality of analgesia in post laparoscopy patients. As such the benefits of conducting the following study will be in investigating:

• A novel intervention to help reduce pain, attenuate post-operative opiate requirements and discomfort for a procedure associated with a moderate to high level of pain level of pain

• The treatment effect of VR so larger RCTs can be subsequently carried out

Should the potential of analgesia be promising post laparoscopy, this will pave the path for testing the device in alternative post-operative scenarios as well.

**14. OTHER INFORMATION**

**14a. Registration**

This trial is not registered with the Australia New Zealand Clinical Trial Registry (ANZCTR).

**14b. Funding and Support**

This project is investigator initiated and is being funded and supported otherwise by Monash University and Monash Health. Additional support is being provided by Biorithm Pte. Ltd. (Singapore) in the form of virtual reality headsets and virtual reality content.

**References**

1. Gerbershagen HJ, Aduckathil S, van Wijck AJ, et al. Pain intensity on the first day after surgery: a prospective cohort study comparing 179 surgical procedures. *Anesthesiology* 2013;118(4):934-44. doi: 10.1097/ALN.0b013e31828866b3 [published Online First: 2013/02/09]

2. Jarrell J, Ross S, Robert M, et al. Prediction of postoperative pain after gynecologic laparoscopy for nonacute pelvic pain. *American journal of obstetrics and gynecology* 2014;211(4):360.e1-60.e8. doi: <https://doi.org/10.1016/j.ajog.2014.04.010>

3. Kehlet H, Jensen TS, Woolf CJ. Persistent postsurgical pain: risk factors and prevention. *Lancet (London, England)* 2006;367(9522):1618-25. doi: 10.1016/s0140-6736(06)68700-x [published Online First: 2006/05/16]

4. Wheatley SA, Millar JM, Jadad AR. Reduction of pain after laparoscopic sterilisation with local bupivacaine: a randomised, parallel, double‐blind trial. *BJOG: An International Journal of Obstetrics & Gynaecology* 1994;101(5):443-46. doi: doi:10.1111/j.1471-0528.1994.tb11920.x

5. Alexander JI. Pain after laparoscopy. *British journal of anaesthesia* 1997;79(3):369-78. [published Online First: 1997/12/09]

6. Ekstein P, Szold A, Sagie B, et al. Laparoscopic Surgery May Be Associated With Severe Pain and High Analgesia Requirements in the Immediate Postoperative Period. *Annals of Surgery* 2006;243(1):41-46. doi: 10.1097/01.sla.0000193806.81428.6f

7. Wiederhold BK, Soomro A, Riva G, et al. Future directions: advances and implications of virtual environments designed for pain management. *Cyberpsychology, behavior and social networking* 2014;17(6):414-22. doi: 10.1089/cyber.2014.0197 [published Online First: 2014/06/04]

8. Dascal J, Reid M, IsHak WW, et al. Virtual Reality and Medical Inpatients: A Systematic Review of Randomized, Controlled Trials. *Innovations in clinical neuroscience* 2017;14(1-2):14-21. [published Online First: 2017/04/08]

9. Garrett B, Taverner T, Masinde W, et al. A rapid evidence assessment of immersive virtual reality as an adjunct therapy in acute pain management in clinical practice. *The Clinical journal of pain* 2014;30(12):1089-98. doi: 10.1097/ajp.0000000000000064 [published Online First: 2014/02/19]

10. Gupta A, Scott K, Dukewich M. Innovative Technology Using Virtual Reality in the Treatment of Pain: Does It Reduce Pain via Distraction, or Is There More to It? *Pain medicine (Malden, Mass)* 2018;19(1):151-59. doi: 10.1093/pm/pnx109 [published Online First: 2017/10/13]

11. Tashjian VC, Mosadeghi S, Howard AR, et al. Virtual Reality for Management of Pain in Hospitalized Patients: Results of a Controlled Trial. *JMIR mental health* 2017;4(1):e9. doi: 10.2196/mental.7387 [published Online First: 2017/03/31]

12. Wu CL, Rowlingson AJ, Partin AW, et al. Correlation of postoperative pain to quality of recovery in the immediate postoperative period. *Regional anesthesia and pain medicine* 2005;30(6):516-22. doi: 10.1016/j.rapm.2005.07.190 [published Online First: 2005/12/06]

13. Knottnerus JA, Muris JW. Assessment of the accuracy of diagnostic tests: the cross-sectional study. *Journal of clinical epidemiology* 2003;56(11):1118-28. [published Online First: 2003/11/15]

14. Hjermstad MJ, Fayers PM, Haugen DF, et al. Studies comparing Numerical Rating Scales, Verbal Rating Scales, and Visual Analogue Scales for assessment of pain intensity in adults: a systematic literature review. *Journal of pain and symptom management* 2011;41(6):1073-93. doi: 10.1016/j.jpainsymman.2010.08.016 [published Online First: 2011/05/31]

**Pre-operative questionnaire:**

1. Do you think the procedure is painful?

YES / NO (**If YES**, **move to question 2**) (If **NO**, move to **question 3**)

1. If yes, please mark on the line below the pain you would anticipate from the procedure


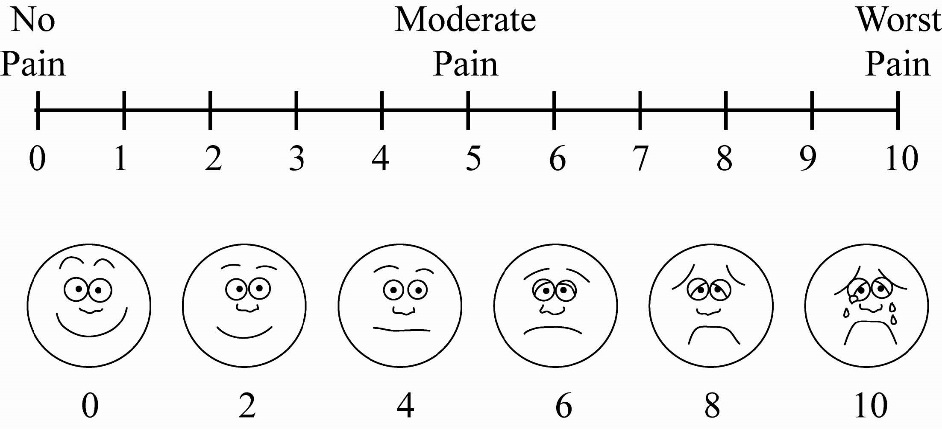


No
Pain at all

Moderate
Pain

Worst Pain

1. Have you found yourself anxious about the procedure?

YES/ NO (**If YES**, **move to question 4**) (If **NO**, move to **question 5**)

1. If yes, please mark on the line below the pain you would anticipate from the procedure


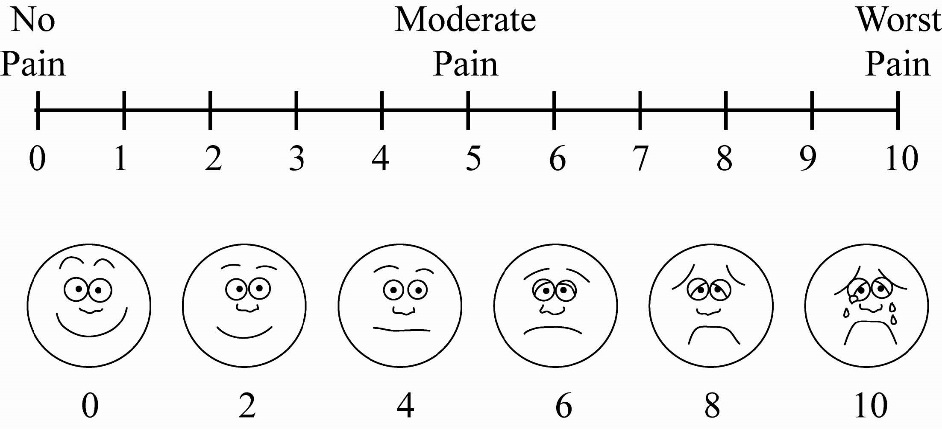


No Anxiety

Moderately Anxious

So Anxious I Want To Run Away

1. Have you ever had an episode of depression in your life? YES/ NO

**Post-intervention questionnaire**

1. Have you ever heard about virtual reality technology for pain relief? YES / NO
2. If YES, from where. If NO proceed to question 4
   1. Books
   2. Internet
   3. Friends
   4. Hospital
   5. Other:
3. How would you rate the experience of utilising the device for the procedure?


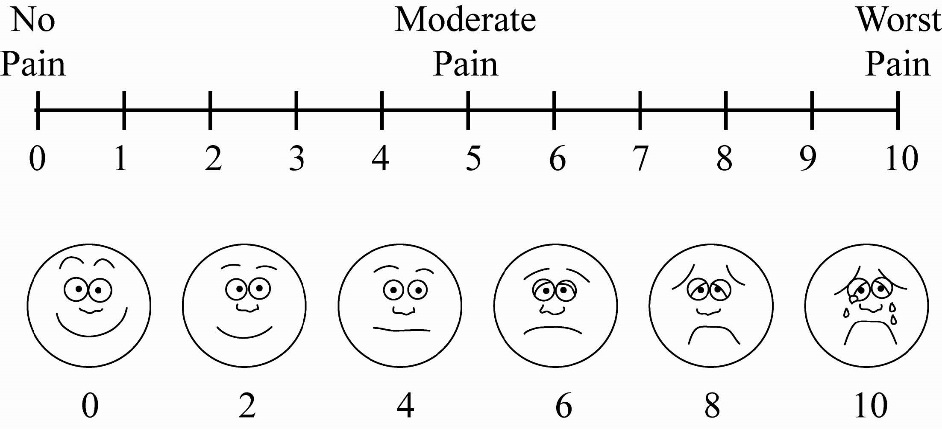


Terrible

Neutral/Ok

Excellent

1. Would you reconsider your decision to have the procedure based on the pain felt?

YES / NO

1. Would you use the device again post laparoscopy? YES / NO
2. Would you recommend the device to your friends for a similar procedure?

YES/ NO

1. In your opinion should VR technology should be used all the time for post-operative pain? YES/ NO
2. Did patient request VR after the trial was ceased? YES/NO
3. Should VR be used in all patients post-laparoscopically? YES/NO
4. Would you use VR again post-laparoscopically as the standard of care in your next procedure? YES/NO

| **Parameters** | **Results for intervention (n=18)** | **Results for control (n=17)** | **p-value** |
| --- | --- | --- | --- |
| **Rate the level of pain you would anticipate from the procedure (11-point NRS)** | 5.44 (± 2.79) | 5.35 (± 2.71) | 0.92+ |
| **Rate the level of anxiety you feel about the procedure (11-point NRS)** | 4.17 (± 3.40) | 2.94 (± 2.51) | 0.24+ |
| **Do you think the procedure is painful?** | | | |
| **Yes** | 16 (88.9%) | 15 (88.2%) | 1.00~ |
| **No** | 2 (11.1%) | 2 (11.8%) |  |
| **Are you anxious about the procedure?** | | | |
| **Yes** | 13 (72.2%) | 12 (70.6%) | 1.00~ |
| **No** | 5 (27.8%) | 5 (29.4%) |  |
| **Have you had previous abdominopelvic surgeries?** | | | |
| **Yes** | 12 (66.7%) | 10 (58.8%) | 0.63# |
| **No** | 6 (33.3%) | 7 (41.2%) |  |
| **Have you ever experienced an episode of depression?** | | | |
| **Yes** | 10 (55.6%) | 9 (52.9%) | 0.88# |
| **No** | 8 (44.4%) | 8 (47.1%) |  |

**Supplementary materials Table A**. Responses to the pre-procedure questionnaire. Mean (± standard deviation), Median (25^th^ percentile to 75^th^ percentile), +Independent samples t-test, # Chi-squared test, ~Fisher’s exact test. * denotes significance.

| **Pain Scores** | **Intervention** | **Control** |
| --- | --- | --- |
| **0 Minutes** | 5.0 (1.75) | 5.0 (4.5) |
| **10 minutes** | 5.0 (2.63) | 4.5 (4.0) |
| **20 minutes** | 4.5 (3.0) | 4.0 (3.75) |
| **30 minutes** | 4.0 (3.13) | 4.0 (3.25) |

**Supplementary materials Table B:** Median (IQR) pain scores of the intervention and control groups at: 0, 10, 20 and 30 minutes.

| **Parameters** | **Results for intervention (n=18)** | **Results for control (n=17)** | **p-value** |
| --- | --- | --- | --- |
| **Have you ever heard about VR for pain relief?** | | | |
| **Yes** | 3 (16.7%) | 4 (23.5%) | 0.69~ |
| **No** | 15 (83.3%) | 13 (76.5%) | 0.69~ |
| **Rate using the VR device (11-point scale)** | 7.97 (± 1.22) | 6.62 (± 1.92) | 0.017+* |
| **Would you reconsider your decision to have the procedure based on the pain felt?** | | | |
| **Yes** | 9 (50.0%) | 8 (47.1%) | 0.86# |
| **No** | 9 (50.0%) | 9 (52.9%) | 0.86# |
| **Would you use the device again post laparoscopy?** | | | |
| **Yes** | 17 (94.4%) | 15 (88.2%) | 0.74• |
| **No** | 1 (5.6%) | 1 (5.9%) | 0.74• |
| **Maybe** | 0 (0.0%) | 1 (5.9%) | 0.74• |
| **Would you recommend the device to friends for a similar procedure?** | | | |
| **Yes** | 18 (100.0%) | 16 (94.1%) | 0.49~ |
| **No** | 0 (0.0%) | 1 (5.9%) | 0.49~ |
| **Should VR be used all the time for post-operative pain?** | | | |
| **Yes** | 13 (72.2%) | 11 (64.7%) | 0.63# |
| **No** | 0 (0.0%) | 0 (0.0%) | 0.63# |
| **Depends on the patient** | 5 (27.8%) | 6 (35.3%) | 0.63# |
| **Did patient request VR after trial was ceased?** | | | |
| **Yes** | 2 (11.1%) | 5 (29.4%) | 0.23~ |
| **No** | 16 (88.9%) | 12 (70.6%) | 0.23~ |
| **Should VR be used in all patient’s post-laparoscopy** | | | |
| **Yes** | 14 (77.8%) | 12 (70.6%) | 0.71~ |
| **No** | 0 (0.0%) | 0 (0.0%) | 0.71~ |
| **Depends on the patient** | 4 (22.2%) | 5 (29.4%) | 0.71~ |
| **Would you use VR again post-laparoscopy as the SOC in your next procedure?** | | | |
| **Yes** | 18 (100%) | 16 (94.1%) | 0.49~ |
| **No** | 0 (0.0%) | 1 (5.9%) | 0.49~ |

**Supplementary materials Table C.** Patient responses to the post-protocol questionnaire. Mean (± standard deviation), Median (25^th^ percentile to 75^th^ percentile), # Chi-squared test, ~Fisher’s exact test, •Fisher-Freeman-Halton exact test. * denotes significance. (SOC, standard of care)

| Patient number | Group assignment | Pre-protocol | 0-10 minutes | 10-20 minutes | 20-30 minutes |
| --- | --- | --- | --- | --- | --- |
| 2 | Intervention | 20 | 0 | 4 | 16 |
| 3 | Intervention | 15 | 0 | 0 | 0 |
| 4 | Intervention | 18 | 0 | 0 | 7.5 |
| 5 | Control | 6 | 3 | 0 | 0 |
| 8 | Control | 6 | 0 | 0 | 7.5 |
| 9 | Control | 0 | 27 | 10.5 | 0 |
| 15 | Control | 32 | 10 | 15 | 3 |
| 19 | Control | 0 | 0 | 0 | 15 |
| 24 | Intervention | 14 | 0 | 0 | 0 |
| 26 | Control | 18 | 6 | 0 | 0 |
| 28 | Intervention | 54 | 6 | 0 | 0 |
| 29 | Control | 12 | 0 | 0 | 0 |
| 31 | Intervention | 30 | 0 | 0 | 0 |
| 32 | Control | 0 | 0 | 7.5 | 0 |
| 33 | Control | 6 | 6 | 0 | 0 |
| 35 | Intervention | 24 | 0 | 0 | 0 |
| 37 | Intervention | 12 | 0 | 0 | 0 |
| 38 | Control | 12 | 0 | 0 | 0 |
| 40 | Control | 12 | 12 | 6 | 7 |
| 41 | Intervention | 15 | 0 | 0 | 0 |

**Supplementary materials Table D.** The number of opioid equivalents administered to each patient who received opioid analgesia: prior to commencing protocol; between 0-10; 10-20; and, 20-30 minutes.
